# Plasma RNA-Based Dual Screening for Early/Extreme Spontaneous Preterm Birth and Early Onset Preeclampsia to Enable Prevention

**DOI:** 10.3390/diagnostics16050660

**Published:** 2026-02-25

**Authors:** Carl P. Weiner, Susan E. Carlson, Hamutal Meiri

**Affiliations:** 1 School of Medicine, Creighton University, Phoenix, AZ 85012, USA; 2 College of Health Solutions, Arizona State University, Phoenix, AZ 85004, USA; 3 University of Kansas Medical Center, University of Kansas, Kansas City, KS 66160, USA; scarlson@kumc.edu; 4 TeleMarpe Ltd., Tel Aviv 6908742, Israel; hamutal62@hotmail.com

**Keywords:** aspirin, disease prevention, docosahexaenoic acid (DHA), plasma RNA, early onset preeclampsia, spontaneous preterm birth

## Abstract

**Background/Objectives**: Preterm birth (PTB) at <33 wks’ gestation annually accounts for more than 60,000 births in the United States and 2 million births worldwide. Of these, spontaneous PTB (sPTB) at <33 wks’ gestation complicates about 1.8% of US births, while early onset preeclampsia (EOP), necessitating delivery at ≤33 wks’ gestation, complicates an additional 0.8% of US births. Current screening is based on medical and pregnancy history and biophysical variables with the goal of sensitizing patients and caregivers to early symptom identification. There is no individual patient risk prediction for sPTB at ≤33 wks’ gestation. We now have preventative therapies for women at high risk for EOP with delivery at <33 wks’ gestation (aspirin) and sPTB at ≤33 wks’ gestation (+/− preterm premature rupture of membranes (PPROMs)) (docosahexaenoic acid (DHA). Both require initiation of therapy by ~16 wks’ gestation for optimal effect, a requirement that current screening options cannot satisfy except for the FMF Combined Test for EOP. Neither do we have either an effective first-trimester screen for sPTB nor a dual screen for both of these major obstetric disorders. **FutureBIRTH**^®^ is a maternal five-plasma RNA panel supported by multiple external validation studies to provide effective dual screening for sPTB at <33 wks’ gestation and EOP with delivery at ≤33 wks’ gestation. Herein, we present the second external validation study for EOP with delivery at ≤33 wks with maternal sampling 12–13 wks 6 d combined with a review of the potential clinical impact of **FutureBIRTH**^®^ on the prevention of sPTB and EOP. **Methods**: Two NIH cohorts totaling 494 women were sampled from 12 to 20 wks. **FutureBIRTH**^®^ marker expression was quantified by polymerase chain reaction (PCR) as in the four preceding external validation studies. **Results**: After appropriate exclusions, there were nine cases (2.4%) of EOP with delivery at ≤33 wks gestation and 370 controls. Of this cohort, 79 (21%) were sampled at <14 wks’ gestation. Two of the nine cases were sampled at <14 wks’ gestation. NAMPT expression at 12–20 wks’ gestation was significantly increased in women destined for EOP with delivery at ≤33 wks’ gestation. Only the addition of diastolic blood pressure improved the predictive accuracy of NAMPT, yielding an AUC of 0.89 with a DR of 89% (8/9). Two cases sampled at <14 wks’ gestation who developed EOP with delivery at ≤33 wks’ gestation were screen positive, and two subjects placed on aspirin before 14 wks’ gestation were screen false positives. **Conclusions**: **FutureBIRTH**^®^ offers dual screening for EOP and sPTB with a sampling window extending down to 12 wks’ gestation, thus enabling the widespread use of preventative therapy.

## 1. Background/Objectives

Early spontaneous preterm birth (sPTB) accounts annually for about 60,000 births in the United States of America (US) and 2 million births worldwide [1,2]. The rate has not changed significantly in over 20 years. These births are associated with the majority of infant and fetal deaths, plus substantial short- and long-term medical complications associated with prematurity, including fetal growth restriction, cerebral palsy and neurodevelopmental disability, creating lifelong health care costs. Of the many proposed pathways leading to preterm birth, sPTB at <33 wks’ gestation complicates approximately 1.8% of US births, while early onset preeclampsia (EOP) necessitating delivery at ≤33 wks’ gestation complicates an additional 0.8% of US livebirths [3].

Screening for either disorder is currently limited. In the US and many other countries, prenatal screening is limited to maternal demographics and medical and pregnancy histories. There is no quantitative individual risk estimate. For example, ACOG [4] recommends serial transvaginal ultrasound cervical length (CL) measurements from 16 to 24 wks’ gestation for women with a singleton pregnancy and a prior sPTB followed by vaginal progesterone with a transvaginal CL < 25 mm. ACOG does not recommend serial CL for women without prior sPTB, but rather visualization of the cervix during a second-trimester anatomy scan, although less than a third of women with short CL in the second trimester experience sPTB. Among women identified with a CL < 25 mm and treated with vaginal progesterone, the prevalence of sPTB is reduced by 33–50% [5]. Other studies indicate the efficacy of vaginal progesterone for women with a short CL (10–20 mm) in the second trimester is similar whether or not there was a prior sPTB [5].

There is currently only one externally validated screening test sold for the identification of women at high risk for PTB (PreTRM^®^, Sera Prognostics, Salt Lake City, UT, USA). Based on a blood sample obtained at 18 ^0/7^–20 ^6/7^ wks’ gestation, several validation studies show ‘fair’ predictive efficacy with an area under the receiver operation characteristic curve (AUC) of 0.68–0.75 [6]. Unfortunately, the test does not separate sPTB from EOP, or sPTB < 37 wks from sPTB ≤ 32 wks’ gestation. In a trial comparing birth outcomes of singleton pregnancies with a second-trimester transvaginal CL > 25 mm, pregnancies judged as low risk for PTB were randomized to PreTRM^®^ screening versus no screening to determine the screening efficacy to predict increased risk of PTB. Screen-positive women were enrolled in a defined risk-reduction protocol that included ultrasounds, frequent office visits, and nursing telephone contacts. The rate of PTB in those women screened and enrolled in the risk reduction protocol was similar to the rate of PTB in unscreened women [7]. In another study of PreTRM^®^, infants whose mothers were screen positive and treated with a risk-reduction protocol had a shorter hospital stay by about 7 days (*p* < 0.05) [8].

For preeclampsia, ACOG [4] recommends a thorough investigation of demography, and medical and pregnancy histories to identify risk factors such as a history of preeclampsia, multifetal gestation, chronic hypertension, diabetes (types 1 and 2), renal disease, phospholipid syndrome, lupus erythematosus (SLE), maternal age > 35 y, high (>29) body mass index (BMI), and a family history of preeclampsia. There is no quantitative individual risk estimate. Though low cost, the detection rate (DR) of this approach is low (54%). ACOG supports the use of low-dose aspirin (81 mg/day) as a preventive measure and recommends initiation at 12–28 wks’ gestation. ACOG does not endorse what is currently the only effective, externally validated first-trimester screen for preterm preeclampsia and EOP developed by the Fetal Medicine Foundation (FMF) and performed at 11–13 wks’ gestation. This test offers a DR of 75% for all preterm preeclampsia (<37 wks’ gestation) and as high as 90% for EOP with delivery at ≤33 wks’ gestation with a 10% false positive rate (FPR). Despite the fact that the FMF screen is superior to the ACOG screen [9] and has been validated in a US population [10], it has not been adopted by US institutes.

It is not just screening that has evolved. After years of investigation, we have early pregnancy preventative therapy for sPTB at ≤33 wks gestation. Docosahexaenoic acid (DHA), an omega-3 fatty acid, lowers the rate of sPTB at ≤33 wks gestation by 65% in first-trimester women deficient in DHA if adherent to supplementation with 1000 mg/d DHA beginning at a mean of ~16 wks gestation [11]. Women with a high baseline level of DHA had the lowest sPTB ≤ 33 wks rates.

These developments highlight the need for widespread screening in the first trimester for women at high risk to develop sPTB. Herein, we describe a novel liquid biopsy test, **FutureBIRTH**^®^, which is capable of screening separately for EOP ≤ 33 wks gestation and sPTB < 33 wks gestation early enough in gestation to enable effective prevention with available therapies. We begin with a new external validation study for EOP ≤ 33 wks gestation, followed by a review of the four preceding **FutureBIRTH**^®^ external validation studies [12,13,14] and the potential it enables for prevention.

## 2. Precision Medicine for Complications of Pregnancy

### 2.1. Materials and Methods

#### 2.1.1. Clinical Methods

Five external validation studies of **FutureBIRTH**^®^ are covered herein. All five studies use the same Methods summarized below [12]. The first 4 studies have been published, and additional details may be found in the referenced publications.

Maternal plasma [4 mL vacuum tubes coated with 7.2 mg K2EDTA] was collected, placed on wet ice, and centrifuged within 60 min. The plasma was aliquoted into 500 µL fractions and stored at −80 °C. All clinical records were extracted contemporaneously and entered into a computerized database. Entries were checked for accuracy on a randomized basis.

Only singleton pregnancies were included. Pregnancy dating used the last menstrual period (LMP) when available, confirmed by an ultrasound performed at ≤20 wks. If the two differed by more than 11 days or there was no LMP, the ultrasound-derived gestation was used. Information was collected on maternal characteristics, including race, ethnicity, education, smoking status, parity, gravidity, previous PTB and the gestation at which it occurred, maternal age, and maternal weight. The blood pressure/mean arterial pressure (MAP) was obtained at the time of sampling.

#### 2.1.2. Laboratory Methods

Laboratory personnel were blinded to all pregnancy outcomes.

##### RNA Extraction

PCF RNA was extracted using a proprietary method. The mean total RNA extracted was 15.9 ± 2.2 µg/mL (±standard deviation).

##### qRT-PCR Assays

mRNA RT: The RNA was diluted with a master mix, including dNTP mix, Omniscript Reverse Transcriptase and Random Primer (Invitrogen, Carlsbad, CA, USA), and converted into cDNA at 37 °C for 60 min per the manufacturer’s instructions.

miRNA RT: miRNA was polyadenylated (Invitrogen NCode miRNA First-Strand cDNA Synthesis Kit, ThermoFisher) and reverse transcribed to generate the first strand of cDNA according to the manufacturer’s protocol.

#### 2.1.3. Preamplification and qPCR

Multiplex qPCR reactions were performed using SYBR Green Supermix (ThermoFisher, Waltham, MA, USA) and the ViiA 7 Real-Time PCR System (ThermoFisher). Probe sets in each reaction well included primers for the biomarker, normalization, and spike genes. The 1 µL RT samples were prepared for the preamplification mix reaction and underwent 12 cycles. The 2 µL of preamplification cDNA samples were diluted into 10 µL PCR reaction mixtures, followed by RT PCR. Threshold cycles (CT values) of qPCR reactions were extracted using QuantStudio™ Software V1.3 (Applied Biosystems, Foster City, CA, USA). The delta-delta CT method was used to calculate RNA expression and then normalized.

#### 2.1.4. Statistics

RNA expression of each of each RNAs, as well as MAP, was typically converted into a multiple of the median (MoM) derived from unaffected pregnancies using either the overall median or a regression if there was an association between expression and either gestational age or maternal weight. The distribution of log MoMs in cases and non-cases was compared using the Wilcoxon Rank Sum Test, and maternal characteristics were compared using a Chi-square test. RNAs and factors with a statistically significant difference (defined as *p* < 0.05 in two-tailed testing) were subjected to logistic regression analyses alone and in combination with a prior PTB for PTB analyses and blood pressure for preeclampsia. Rarely, RNA expression levels fell below detection. In those instances, separate regression equations were derived for the detected RNA co-marker. Each regression yielded an AUC to obtain a measure of performance across all possible classification thresholds.

A regression equation was used to derive a probability, *p*, where *p* = y/(1 − y), y = ex, and x is a linear function of log MoMs and maternal characteristics. Among controls, the 90th, 80th and 70th percentile of *p* was used to determine the observed DR for three fixed FPRs (10%, 20% and 30%) since an acceptable FPR could vary if the cost of misclassification was part of model selection. The number of cases per clinical group varied by case definition (PTB < 37 wks, sPTB < 33 wks, preeclampsia and EOP ≤ 33 wks), but the total sample size (case + non-case) remained constant.

### 2.2. Results

#### 2.2.1. External Validation Studies

##### EOP with Delivery at ≤33 Wks

This was the second external validation study for EOP with delivery at ≤33 wks’ gestation (Table 1) and based on a prospective cohort of 494 singleton pregnancies sampled at 12–20 wks’ gestation (413 were participants in the ADORE Trial [11] and 81 participants in the Predisposition to Develop Preeclampsia Trial [15]). The original goal was to screen for both sPTB and EOP, but surprisingly, there was only one case of sPTB at <33 wks’ gestation after the anonymous diagnosis codes were revealed. After appropriate exclusions (lost to follow-up, study withdrawal, preterm delivery at <37 wks and >33 wks, term preeclampsia, induction of labor for fetal anomalies), there were nine cases (2.4%) of EOP with delivery at ≤33 wks’ gestation and 370 controls. Of this sub-cohort, 79 (21%) were sampled at <14 wks’ gestation. Two of the nine cases were sampled at <14 wks’ gestation. There were two other women sampled at <14 wks’ gestation who were started on aspirin well before enrollment (aspirin dose and reason for starting not clearly recorded). Other variables recorded in both trials varied in consistency and granularity, and as a result, none were considered in the analysis. All subjects provided informed written consent, and the methods used were identical to prior published studies [12,13,14].

NAMPT expression at 12–20 wks’ gestation was the only RNA significantly increased in women destined for EOP with delivery at ≤33 wks’ gestation. Only the addition of diastolic pressure at sampling improved the predictive accuracy of NAMPT, yielding an AUC of 0.89 with a DR of 89%. The two cases sampled at <14 wks’ gestation who developed EOP with delivery at ≤33 wks’ gestation were screened positive, extending the **FutureBIRTH**^®^ sampling window for EOP down to 12 wks’ gestation. The two subjects placed on aspirin at <14 wks’ gestation were screened false positives.

We compared the aforementioned study to the first external validation study for EOP with delivery at ≤33 wks’ gestation based on a different prospective cohort of 305 pregnancies sampled from 16 to 20 wks’ gestation [12,13] (Table 2). There were six cases of EOP with delivery at ≤33 wks’ gestation (1.9%). NAMPT and APOA1 sampled from 16 to 20 wks’ gestation were individually predictive of EOP with delivery at ≤33 wks’ gestation. The predictive algorithm for EOP with delivery at ≤33 wks’ gestation included expression of the two RNAs, parity and MAP and yielded an AUC of 0.96 with a DR of 100%. The 95% CI for the AUC achieved in this second external validation study for EOP overlapped the AUC of the first external validation study for EOP based on samples obtained between 16 and 20 ^0/7^ wks’ gestation [13].

##### sPTB < 33 Wks

**Figure 1:**
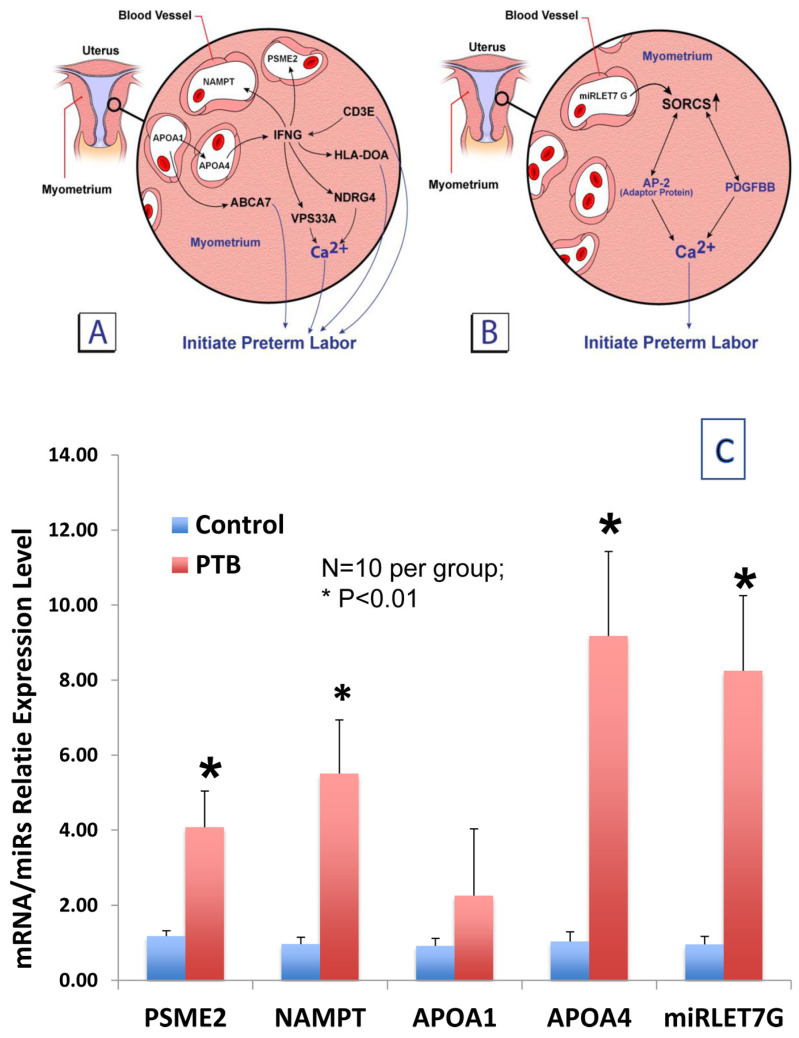
(**A**,**B**): The 5 potential RNA markers were selected after an in silico enquiry identified them as interacting directly or indirectly with 7 preterm initiator genes. (**C**): Confirmation of q-rRT-PCR performed for the 5 RNA markers using the same 20 patient Discovery samples. Four of the 5 potential sPTB markers were confirmed. APOA1 just missed significance but has since remained in the panel. BJOG 2021; 128:1870-80.

**FutureBIRTH**^®^ began with the discovery of differentially expressed (DE) PCF RNAs predictive of sPTB at <33 wks’ gestation [12]. A total of 297 DE RNAs for sPTB at <33 wks’ gestation were identified. Rather than rely on preconceived disease theories or the magnitude of differential expression, we compared in silico the DE PCF RNAs to a list of myometrial DE RNAs from laboring and nonlaboring women at term and <33 wks’ gestation [16]. Five PCF RNAs interacted with seven myometrial Preterm Initiator genes (Figure 1A,B) [12]. These five RNAs were overexpressed in the placenta of women with sPTB at <33 wks and increased intracellular Ca^2+^ transport and the frequency of cell contraction in immortalized human pregnant myometrial cells.

The first external validation study of the RNA panel for sPTB at <33 wks was a case-controlled study published with the original Discovery study (Table 3) (12). Forty women free of comorbidities and not part of the Discovery investigation were tested between 16.0 and 20.0 wks’ gestation. Gestation at delivery was 26.5 ± 2.6 wks for sPTB and 40.1 ± 0.9 wks for controls. Reviewers required the validation analysis to compare median expression for each RNA individually, and each was shown to be a significant predictor of sPTB at <33 wks’ gestation. Logistic regression analysis was also performed to derive an equation for each of the confirmed RNAs, including those with missing values. Each individual RNA AUC exceeded 0.98 with a DR of 100%. Combining all equations into a single panel reached an AUC of 1.00.

The second external validation study for sPTB at <33 wks’ gestation (Table 4) was performed on the same prospective cohort of 305 pregnancies sampled from 16 to 20 wks’ gestation and used for the EOP study [13]. PSME2 and Let 7g were the best performers, and the predictive algorithm for sPTB at <33 wks’ gestation included expression, history of prior preterm birth, and race, and yielded an AUC of 0.83 with a DR of 77%. The algorithm also identified additional cases of sPTB from 33 to 36 wks’ gestation with an AUC of 0.77 for all sPTB at <37 wks’ gestation with a DR of 73%.

The third external validation study for sPTB at <33 wks’ gestation (Table 5) was a unique case-controlled study of 60 nulliparous women sampled in the first trimester (12 to 13 wks’ gestation) [14]. The predictive algorithm included RNA expression and the limited descriptive data available (crown rump length, maternal age, weight, race, and tobacco use). Again, PSME2 was a significant predictor of sPTB at <33 wks’ gestation with an AUC of 0.71, coupled with demographic variables. However, APOA1 replaced Let 7g, which, combined with demographics, yielded an AUC of 0.79 with a DR of 79%. Combining PSME2 and APOA1 did not improve the AUC above that of APOA1 alone in these first-trimester samples. Despite the modest sample size, the 95% CI for the AUC overlapped the AUC for the prediction of sPTB at <33 wks’ gestation achieved in the second validation study conducted on 16 to 20 wks’ gestation samples, thus extending the validated sampling gestation for **FutureBIRTH**^®^ down to 12 wks’ gestation.

### 2.3. Explaining the Dual Efficacy of FutureBIRTH^R^ to Predict Both sPTB and EOP

The panel of five RNAs was selected based on their association with DE myometrial RNA in women with sPTB at <33 wks’ gestation [16]. Yet, we noticed during the initial cohort analysis that two of the five RNAs appeared more predictive of EOP with delivery at ≤33 wks’ gestation than sPTB at <33 wks’ gestation. The subjects comprising the PCF RNA Discovery investigation strictly excluded preeclampsia/hypertension. However, a review of the records from subjects providing myometrial samples revealed that most nonlabor samples from deliveries < 33 wks’ gestation were from women with EOP that was unresponsive to oral antihypertensives. Moreover, several cases of sPTB at <33 wks’ gestation also had hypertension.

It seems our assumption in the late 1990s and early 2000s that sPTB and EOP were unrelated disorders may be wrong. Each of the five PCF RNAs increases intracellular Ca^2+^ transport in smooth muscle cells, and sPTB and EOP share abnormal smooth muscle activity. Cell-specific targeting of PCF RNAs is determined by the RNA’s extracellular transporter, not by the RNA. In addition, 8–12 wks’ gestation coincides with a period of rapid placental development, especially of the villous tree [17]. A change in transporter should not be surprising.

### 2.4. Why Has No Other PCF RNA Test Been Externally Validated for Any Disease or Complication Repeatedly Despite Extensive Effort?

No DE PCF RNA identified in any pregnancy Discovery study has been shown to be unique to pregnancy. The first reports of PCF RNA extraction utilized EDTA plasma and the Trizol LS Reagent. Concurrently, the first commercial plasma RNA extraction kits were being introduced and were followed by more specialized versions around 2010 that targeted specific types of RNAs, for example, microRNAs. These tools were rapidly adopted by laboratories as time and cost savers while facilitating efforts to study specific RNA types. Virtually all peer-reviewed PCF RNA Discovery and Validation studies relied on one or more of these extraction kits. Over time, it has become clear that kits vary in the profile of RNA extracted and RNA prevalence [18,19,20,21]. It is unclear whether a given kit extracts the same profile of RNA from each sample or the same sample. It is clear that the total RNA quantity extracted by these kits is low, with yields (when provided in publications) typically ranging from 50 to 500 ng per milliliter of plasma.

The plasma RNA extraction developed in our laboratory yields a mean of 15.7 ug/mL plasma (range 3.3–88.0 ug/mL plasma) [22]. The extraction includes coding and noncoding RNAs. This quantity of total plasma RNA means that the results of similar studies of the plasma transcriptome using commercial kits are based on 1% or less of the total plasma RNA in the sample, increasing the likelihood of a highly variable RNA extraction and, as a result, making external validation virtually impossible. Alternatively, it is possible that by reaching the microgram range of extracted RNA overcomes potential inaccuracies introduced by very low yields of RNA near the limit of detection. All **FutureBIRTH**^®^ studies have utilized our laboratory’s proprietary extraction.

## 3. Discussion

The advantage of **FutureBIRTH**^®^ is first-trimester dual screening for both EOP with delivery at ≤33 wks’ gestation and sPTB at <33 wks’ gestation. The five external validation studies demonstrate the potential of these plasma RNA markers to provide accurate and early pregnancy prediction of the two most common causes of PTB at ≤33 wks. The consistent performance of the RNA panel on samples from different populations suggests that future larger studies are highly likely to also perform within the 95% CI. Further, it is likely the addition of demographic/clinical variables, such as prepregnancy diabetes, chronic hypertension, etc., will further enhance screening accuracy.

The failure worldwide to lower the rates of early/extreme PTB (at ≤33 wks’ gestation) with existing interventions reflects our limited understanding of the causes of PTB. The current clinical focus on demographics, medical and family histories, and mid-second-trimester markers of various kinds is the product of an unfulfilled need for physicians to provide impactful care. Neonatal survival is the beginning, not the end of prematurity’s adverse effects. A recent population-based study documented the impact of PTB on the first five years of life [23]. The risk of rehospitalization was highest at ages 6–12 mo across all PTB groups compared to term. Children born preterm had increased risks of respiratory, cardiac, endocrine, gastrointestinal, hematological, kidney, neurodevelopmental, and sleep disorders. Further, prescriptions for antibiotics, corticosteroids, diuretics, thyroid hormone, and bronchodilators were increased among preterm children compared to children born at 39–41 wks.

The success of the first-trimester screening with the FMF Combination test and the use of aspirin for the prevention of preterm preeclampsia and EOP requiring delivery at ≤33 wks’ gestation was a large step forward. However, there remains an even larger need for screening and prevention of sPTB at <33 wks’ gestation. Liquid biopsy using the plasma transcriptome appears a potential solution, as there have been hundreds of peer reviewed Discovery type studies covering a multitude of disorders since Lo [24] first reported PCF RNA in 1999, and Poon et al. reported in 2000 fetal PCF RNA in maternal plasma [25].

**FutureBIRTH**^®^ offers good to excellent screening efficacy (AUCs = 0.83–0.96), detecting by 12 wks’ gestation in women at greatest risk with DRs ≥ 80% for these two expensive, severe pregnancy disorders. The fact that **FutureBIRTH**^®^ can effectively prognosticate these two disorders independently before 14 wks’ gestation suggests we now shift our primary screening focus away from 18 to 22 wks’ gestation to the first trimester. It also questions the practice of continuing to rely on screening tests performed after 16 wks gestation when it is too late for effective prevention with the available therapies, even if the screening test has AUCs and DRs similar to **FutureBIRTH**^®^.

The predictive values of **FutureBIRTH**^®^ for samples obtained at 12–20 wks’ gestation proved similar in external validation studies of five different populations for sPTB at <33 wks’ gestation and four populations for EOP with delivery at ≤33 wks’ gestation, including multiple races and ethnic groups. This does not mean gestational age is irrelevant. In blood samples obtained from 16 to 20 wks’, PSME2 plus Let 7g and NAMPT plus APOA1 were, respectively, good to excellent predictors of sPTB at <33 wks’ gestation and EOP with delivery at ≤33 wks’ gestation. However, in the first trimester (12–13 ^6/7^ wks’ gestation samples), APOA1 replaced Let 7g as the best predictor of sPTB at <33 wks’ gestation, and NAMPT alone proved from 12 wks to 20 wks’ gestation an effective predictor of EOP with delivery at ≤33 wks’ gestation. Future studies need to focus on the first trimester since fewer than 200 first-trimester samples have been studied to date. It is important to recognize that PCF RNAs are different from tissue RNAs. Plasma RNAs are typically associated with one of several extracellular vehicles (EVs) that not only protect the RNA from plasma RNases but also provide a mechanism for specific cell type targeting. We know that from 8 wks to 12 wks the placenta is rapidly maturing; a change in the EV for a given RNA being produced by the placenta, resulting in a new cell target, should not be surprising [17].

There are numerous discovery-level investigations for a large variety of disorders, yet no PCF RNA diagnostic based on expression is currently offered in clinical practice. Likewise, multiple laboratories have sought PCF RNA markers over the last decade for preeclampsia. Mirvie Inc. (South San Francisco, CA, USA) published three major Discovery investigations between January 2022 and April 2025 based on internal validation, including over 13,500 pregnancies [26,27,28]. The sampling gestational age window for each paper in chronological order was 16–27 wks, 5–16 wks and 17.5–22 wks’ gestation. The three publications report a total of 41 potential RNA markers (n = 7, 18, and 16), but surprisingly, only one plasma RNA was identified in two of the studies—PAPPA2 RNA was identified in the first and third studies that happen to overlap gestational sampling windows. Mirvie has launched a commercial screening test (Encompass^TM^) in the US for women who fulfill the following requirements: a. 18–22 wks’ gestation; b. ≥35 yo; c. no pre-existing high-risk conditions for preeclampsia; and d. an RNA test result from Mirvie considered positive based on the expression of placental-associated RNA markers for pregnancy hypertensive disease. These restrictions raise questions. Why delay screening until a gestational age when the only available preventative therapy for the disorder, aspirin, has low efficacy? In the US, about 20% of pregnancies are in women aged 35 or older, and while these pregnancies have a higher rate of PTB, the majority of these women are low risk by non-test criteria. Data from 2021 to 2023 show that the PTB rates rise with age, the highest among women aged 40 and older, not 35 [29]. Another molecular biology laboratory [30] also reported in 2025 a prospective cohort of 9586 women sampled longitudinally in three windows similar to Mirvie: 9–14 wks, 18–28 wks, and either after 28 wks’ gestation or when the women developed clinical preeclampsia. They identified 36 first-trimester PCF RNAs as potential markers for EOP diagnosed at <34 wks’ gestation and 87 second-trimester PCF RNAs potentially predictive of EOP at <34 wks’ gestation. Not one of the DE RNAs was repeated in the first two sampling periods, nor was there one RNA that overlapped with the Mirvie-suggested RNAs obtained at similar gestational ages. The lack of reproducibility held true for the list of RNAs predictive of late-onset preeclampsia. Finally, there are also no published external validations, the gold standard, for any of these proposed markers. Clearly, neither matches the efficacy of the FMF Combination test for preeclampsia performed at 11–13 wks’ gestation (discussed subsequently).

**FutureBIRTH**^®^ AUCs and DRs for sPTB at <33 wks are also superior to PreTRM^®^, fetal fibronectin, and transvaginal/transabdominal ultrasound cervical length (Table 6). No alternative screening test is clinically useful in the first trimester and thus has low potential to change clinical outcome. For women who miss the first trimester sampling window for preventative therapy, **FutureBIRTH**^®^ screening efficacy in the early second trimester is still superior to all of the alternatives. As for EOP, **FutureBIRTH**^®^ AUCs and DRs are similar to the FMF test, which was the basis of multiple successful aspirin trials. For women who miss the first trimester window, **FutureBIRTH**^®^ for EOP is still effective for women up to 20 wks gestation and has higher performance than Encompass^TM^.

Seafood is an important source of the omega-3 fatty acids, DHA and eicosapentaenoic acid (EPA). After observing a longer duration of gestation in Faroe Islanders compared to Danes who consumed less omega-3 fatty acids, Olsen et al. suggested it was due to differences in their omega-3 fatty acid intake [34]. In 2000, Olsen et al. [35] published a clinical trial with DHA and EPA in women considered at high risk for PTB. Participants were enrolled in 19 European hospitals and provided a fish oil supplement containing 900 mg/day DHA and 1200 mg/day EPA. Supplementation was associated with a reduced rate of all recurrent PTB from 33% to 21% (a 36% decline) and reduced all PTB at ≤33 wks’ gestation from 14.9% to 10.6% (a 29% decline).

Eighteen years later, a Cochrane Review [36] based on 70 randomized controlled trials concluded there was strong evidence that omega-3 fatty acid supplementation reduced the risk of all PTB by 11% (27 studies) and PTB at ≤33 wks’ gestation by 42% (8 studies). The authors concluded the results were driven by studies providing ≥500 mg/d of DHA. Supplementation had no significant effect on rates of preeclampsia or any other adverse maternal outcome, suggesting the effect of DHA was solely on sPTB. *However, key questions remained unanswered, particularly ‘when’, ‘how much’ and ‘for whom’.*

Two large trials with the aim of reducing PTBs at ≤33 wks’ gestation with high-dose DHA were published after the 2018 Cochrane Review. Both the US ADORE trial [37] and the Australian ORIP trial [38] were performed after prenatal vitamins containing DHA were being marketed in the US, Australia and other countries. The use of DHA-containing supplements prior to enrollment in about half of the ADORE subjects may explain why only women with low DHA on enrollment (also about half of enrollees) benefited from assignment to 1000 mg/d vs. 200 mg/d with fewer PTBs at ≤33 wks’ gestation (2.0% vs. 4.1% (i.e., a 51% decline) [37]. A secondary analysis revealed the rate of all PTBs decreased by 26% (from 11.0% to 8.1% (PP = 0.95). While there was no benefit in the ORIP trial from high-dose supplementation (800 mg/d DHA + 100 mg/d EPA vs. placebo) [38], a secondary analysis revealed the subjects with the lowest omega-3 status on enrollment (885 of 5544) benefited [39]. The results of these trials were used to update the 2018 Cochrane Review, and the original conclusion held, showing a 12% decrease in all PTBs and a 35% decrease in PTBs at ≤33 wks’ gestation [40]. *Both trials found that average levels of DHA or omega-3 fatty acid in the first few months of pregnancy substituted for high-dose DHA supplementation to reduce PTB at ≤33 wks’ gestation.*

In the ADORE trial [37], half the women had low DHA on enrollment. Their PTB rate on 200 mg was 82.5% higher than women who started the trial with normal to higher DHA status (14.6% compared to 8.0%). The PTB at ≤33 wks’ gestation on low-dose DHA was more dependent on baseline status, with a rate of 4.8% in those with a low baseline DHA compared to 1.6% in those with higher baseline DHA (a 67% reduction).

**Figure 2:**
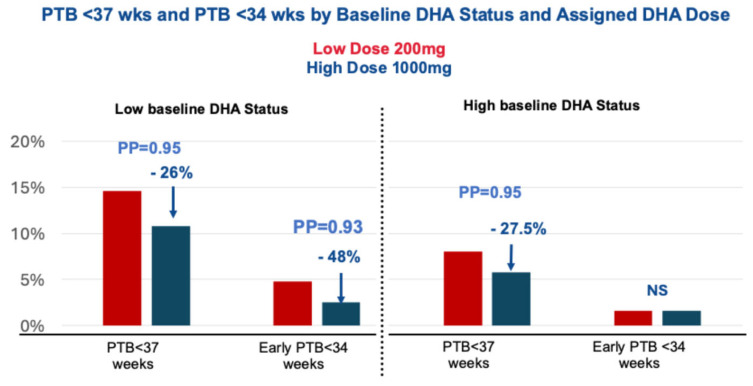
Results adapted from Carlson et al. Higher dose docosahexaenoic acid supplementation during pregnancy and early preterm birth: a randomized, double-blind, adaptive-design superiority trial. Adapted from EClinMed 36 (2021) 100905 [37]. Low baseline status is defined as <6% RBC-PL-DHA.

Women who received a high dose DHA (1000 mg/d) had a lower rate of all PTBs in the low DHA baseline group (14.6% to 10.8%, a 26% decline) (posterior probability or PP = 0.95) [37]. Only women with low DHA status at enrollment benefited from 1000 mg/d DHA to reduce the rate of PTBs at ≤33 wks’ gestation in the low DHA baseline group by 48% from 4.8 to 2.5% (PP = 0.93) but had no further effect in the high baseline group (Figure 2) [37].

**Figure 3:**
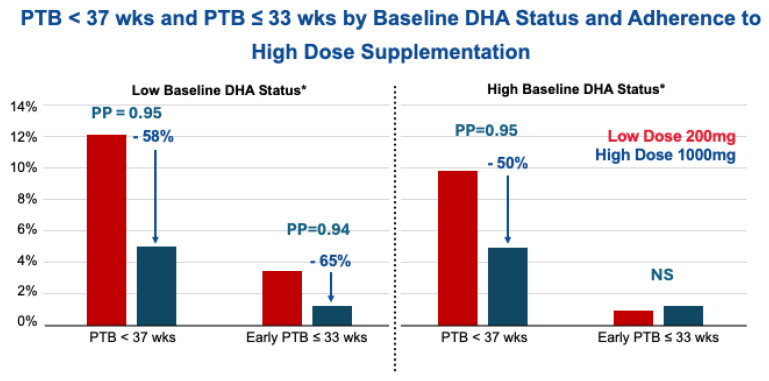
* Baseline < 6.2% or ≥6.2% RBC-PL-DHA used to define low and high baseline; postpartum ≥ 8% RBC-PL-DHA used to define adherence. Results adapted from Carlson et al. [37].

With compliance, 1000 mg/d produced a 58% reduction in all PTBs (12.0% to 5%) for the low baseline group, 50% (9.8 to 4.95%) for the high baseline group (both PP = 0.95), and an impressive 65% reduction (from 3.45% to 1.20%) in PTBs at ≤33 wks’ gestation in the low baseline group but no effect in the high baseline group (Figure 3) [37].

*Women who began a prenatal supplement with DHA before enrollment had the lowest rates of PTBs and PTBs at ≤33 wks, supporting the need for early supplementation.* DHA was initiated at a mean of 16 wks’ gestation, and there was no enrollment after 20 wks’ gestation, suggesting supplementation is best if begun at least in the first half of pregnancy [41].

Does maternal blood DHA need to be measured in the laboratory? Carlson et al. questioned whether DHA intake could be a practical tool to determine who might benefit from high-dose DHA supplementation (800 or 1000 mg/d vs. 200 mg/d) [42]. They combined the results of the ADORE and the PANDA Trials [41], both of which utilized a 7-question DHA-Food Frequency Questionnaire (DHA-FFQ) in parallel with laboratory testing of DHA. Participants consuming <150 mg/d DHA at baseline, if randomized to the 1000 mg/d DHA, had a 28% lower PTB rate (8.6% vs. 12%) and a 58% lower rate of PTB at ≤33 wks’ gestation (1.5% vs. 3.6%) [42]. In contrast, women consuming >150 mg/d at baseline had lower rates of PTBs and PTBs at ≤33 wks’ gestation compared to those consuming <150 mg/d, and there was no additional effect of higher 1000 mg/d DHA on the >150 mg/d women.

The now proven efficacy of DHA to prevent sPTB at ≤33 wks’ gestation in high-risk women is a crucial clinical development. In 2022 and 2024, the US and Australian groups published guidelines for omega-3 fatty acid intake to reduce PTB [43]. Both emphasized the need for women with either a low DHA baseline or low DHA intake to add DHA plus EPA or a DHA supplement of 1000 mg/d or a supplement between 600 and 1000 mg/d, respectively, to their regimen. The ISSFAL statement [44], written by the Australian ORIP team, follows their study protocol and recommends only women with low blood DHA take 1000 mg/d of DHA. These guidelines were published in ACOG MFM in 2025 [43] and are now backed by at least seven professional organizations: European Board & College of Obstetrics and Gynaecology; European Academy of Paediatrics; European Society for Paediatric Research; Asia Pacific Health Association (Pediatric-Neonatology Branch); International Society for Developmental Origins for Health and Disease; Child Health Foundation (Stiftung Kindersgesundheit); and European Foundation for the Care of Newborn Infants Parent Organization. The guidelines recommend:

(a) Daily intake of at least 250 mg/d DHA + EPA in women of child-bearing age from diet and/or supplements.

(b) Assess DHA status early in pregnancy either by intake or blood level.

(c) Document DHA status both verbally and in writing.

The guidelines recommend women with good DHA status be instructed to increase their intake of DHA by 100 to 200 mg/d, and for women with low intake or status, to increase their intake by 600 to 1000 mg/d of DHA + EPA (recognizing it is DHA and not EPA that is associated with reduced sPTB, so no more than 100 mg/d in the supplement should be derived from EPA).

Progesterone has also been under study for decades as a therapeutic to prevent primary and or recurrent spontaneous PTB. There is no widespread consistency on the need for a prior sPTB, the need for CL shortening or the degree of CL shortening necessary to trigger treatment, nor the gestational age for optimal screening, the route of administration, nor the dose of progesterone. Ninety percent of women with sPTB at <34 wks’ gestation have no prior history of sPTB, leaving the obstetric caregiver to focus on medical history plus CL measured at 18–22 wks’ gestation. Unfortunately, the AUC of a transvaginal CL at 16–22 wks’ gestation for the prediction of sPTB at <37 wks’ gestation using a cutoff of ≤25 or 20 mm is only 0.53 and 0.51 [31]. An AUC < 0.60 is generally considered a failed test. Perhaps similar to the evolution of the aspirin story for the prevention of EOP, the variability in progesterone clinical trial outcomes reflects the lack of an effective individual patient screen to provide a homogenous pool of subjects. The availability of a more effective screening tool might bring clarity to the efficacy of progesterone.

Absent a prior sPTB, the SMFM currently recommends [43] the following:

- All CL measurements guiding therapy should be transvaginal and performed at 18–22 wks’ gestation at the anatomic scan.
- A midtrimester CL of ≤25 mm is considered short in women with a singleton pregnancy but no prior sPTB, and those with a CL of ≤20 mm should be prescribed vaginal progesterone.
- In such women with a CL of 10–25 mm, cerclage should not be performed absent cervical dilation, nor should a pessary be prescribed.

In women with a prior sPTB, ACOG currently recommends [6] the following:

1. Serial vaginal CL (every 1–4 wks’) at 16 ^0/7^–24 ^0/7^ wks’ gestation.
2. Offer vaginal progesterone for asymptomatic women with a prior sPTB.
3. Consider cerclage with a CL of <25 mm (vs. progesterone if not already on it).

Despite the current recommendations, vaginal progesterone appears to work equally well in women with a CL of 10–20 mm at 18–22 wks but no history of PTB [5].

The ASPRE study [45] was a clinical leap forward, combining 11–13 wks screening for preterm preeclampsia (<37 wks gestation) and EOP with delivery at ≤33 wks’ gestation with prevention using 150 mg aspirin taken nightly between 12 and 36 wks gestation in women with a 1/100 or greater risk for preterm preeclampsia. The rate of preterm preeclampsia was reduced by 62% and that of EOP with delivery at <33 wks’ gestation by 82% [46]. The study used the FMF model for predicting preeclampsia, which considers the following: (1) a quantitated prior risk calculated from demographic information (maternal age, BMI, ethnicity), the medical history (chronic diabetes, cardio-vascular complications, phospholipid syndrome and lupus erythematosus (SLE)), pregnancy history (parity, previous preeclampsia or FGR, family history of preeclampsia) and mode of conception, and (2) a quantitated posterior risk combining the above with MAP and the UtA-PI along with biochemical markers (placental growth factor (PlGF) and pregnancy associated placental protein A (PAPP-A) [47]. Markers are converted to MOMs, and the risk is calculated using the competing risk model. The DR for all preterm preeclampsia requiring delivery at <37 wks’ gestation was 75%, and the DR for EOP necessitating delivery at <33 wks’ gestation was 90%, both with a 10% FPR [48].

The ASPRE study was subsequently validated in multiple geographic and ethnic groups, including the Far East [39], US [10], Italy, Australia [49], Switzerland and Norway, among others. A cost/benefit analysis found screening followed by treatment with low-dose aspirin was cost-effective, mainly by a reduction in NICU admissions and days [50]. And yet, it has not translated to a universal method outside certain medical centers in the UK and limited regions in Australia and Canada. Denmark is conducting a national validation with the intent to launch nationwide [51].

ACOG and NICE have not adopted the ASPRE approach, concluding the results are not sufficiently effective despite the comparative analysis of Poon et al. demonstrating higher efficacy of the ASPRE method compared to either the ACOG or NICE [52], and the randomized SPREE study, which compared NICE and ASPRE screening conducted by Tan et al. [53].

Aspirin was initially tested to prevent preeclampsia based on its ability to block thromboxane-induced vasoconstriction [54]. Indeed, a recent study demonstrated that aspirin dilates isolated human uterine arteries [55], potentially improving oxygen and nutrient supply to the placenta and fetus. Additional studies indicate that prophylactic aspirin is associated with a reduced UtA-PI, potentially providing a way to monitor treatment efficacy [32].

The timing of aspirin use has been debated for years. ACOG suggests initiation any time between 12 and 28 wks’ gestation with a preference for earlier use. Bujold et al. found [33] the efficacy of aspirin in preventing preeclampsia was limited when started after 16 wks’ gestation. The optimal dose has also been debated, given an earlier study from Sibai et al. [56] reporting an increased risk of placental abruption with aspirin. However, a meta-analysis [57] concluded that daily aspirin of ≥100 mg and initiated at ≤16 wks’ gestation, rather than at >16 wks’ gestation, may actually decrease the risk of placental abruption or antepartum hemorrhage. In another meta-analysis [58], a dose of ≥100 mg/day was required to significantly reduce the rate of preeclampsia, with the highest efficacy obtained using 150 mg/day.

In summary, clinicians now have two validated therapies, one capable of preventing more than 60% of sPTBs at <33 wks and one capable of preventing more than 80% of EOP with delivery at ≤33 wks’ gestation when initiated before 16 wks’ gestation. Unfortunately, there is no clinically deployed screening test capable of identifying women at high risk for sPTB at <33 wks, and the only such validated test for EOP with delivery at ≤33 wks’ gestation is having trouble with adoption. **FutureBIRTH^®^** offers the potential of effective first-trimester screen for both disorders should larger studies focused on first-trimester screening confirm the already performed external validation studies.

## 4. Conclusions

These two pregnancy disorders are worldwide problems. First-trimester testing is crucial if therapy is to be begun by 16 wks’ gestation, and screening for only one of the two disorders will not alter the rate of the other. **FutureBIRTH**^®^ is the first validated answer to the afore challenge. It is based on PCR technology that is widely available in high, middle and low resource countries, and the therapies are available at low-to-moderate cost. The **FutureBIRTH**^®^ kit model allows for local performance by skilled medical laboratories, minimizing cost and maximizing availability to all countries. Prior detailed investigations indicate that the impact of widespread screening and the resulting decrease in PTB will significantly lower morbidity, mortality, and overall health care costs [59].

**FutureBIRTH**^®^ is the only PCF RNA panel whose efficacy has been repeatedly confirmed in multiple external validation studies. It uniquely detects by 12 wks‘ gestation women at greatest risk for the two most expensive pregnancy disorders and does so with good to excellent AUCs and DRs of >80%. Based on existing studies, routine prenatal screening has the potential to reduce the early/extreme US PTB rate from 2.6% to 1.0%. Any significant decline would be highly likely to be cost-effective in all health care systems.

## Figures and Tables

**Table 1:** External validation study predicting EOP with delivery at ≤33 wks’ gestation #2.

Outcome	Model Variables	AUC [95% CI]	DR (%)
EOP with delivery at ≤33 wks	NAMPT, MAP	0.89 [0.81–0.97]	89

**Table 2:** External validation study predicting EOP with delivery at ≤33 wks’ gestation #1.

Outcome	Model Variables	AUC [95% CI]	DR (%)
EOP with delivery at ≤33 wks	NAMPT, APOA1, parity, MAP	0.96 [0.92–0.996]	100
All preeclampsia	NAMPT, APOA1, parity, MAP	0.82 [0.72–0.91]	71

**Table 3:** External validation study predicting sPTB at <33 wks’ gestation #1.

Marker	Cases	Controls	Values ≥ Median		*p*-Value
			Cases	Controls	
PSME2	20	20	19 (95%)	1 (5%)	<0.0001
NAMPT	13	11	12 (92%)	0 (0%)	<0.0005
APOA4	18	20	18 (100%)	1 (5%)	<0.0001
LET-7g	20	20	19 (95%)	1 (5%)	<0.0001

**Table 4:** External validation study predicting sPTB at <33 wks’ gestation #2.

Outcome	Model Variables	AUC [95% CI]	DR (%)
sPTB at <33 wks	PSME2, Let 7g, prior PTB, race	0.83 [0.74–0.92]	77
sPTB at <37 wks	PSME2, Let 7g, prior PTB, race	0.77 [0.70–0.83]	73

**Table 5:** External validation study predicting sPTB at <33 wks’ gestation #3.

Outcome	Model Variables	AUC [95% CI]	DR (%)
sPTB at <33 wks	APOA1 + CRL, MA, MW, race, tobacco	0.79 [0.66–0.91]	79

**Table 6:** Screening Tests for sPTB and Early Onset Preeclampsia.

Test Name [References]	Patient Inclusions /Exclusions	Earliest Test Use (wks)	Screening Target and Validation	AUC	DR (%)
FutureBIRTH^®^ [12,13,14]	None	12	sPTB at <33 wks (External validation)	0.79–0.83	77–79
Fetal fibronectin [31]	None	16–22	All PTB at <37 wks (External validation)	0.51–0.52	3–15
TV Cervical Length [31]	None	16–22	All PTB at <37 wks (External validation)	0.53	4–8
PreTRM^®^ [6,7,8]	None	19	All PTB at <37 wks (Internal and external validations)	0.68–0.75 PTB at <37 wks 0.76 PTB at <32 wks	75
					
FutureBIRTH^®^ [13]	None	12	EOP delivery at ≤33 wks (External validation)	0.89–0.96	89–100
FMF Combined Test [32,33]	None	11	EOP with delivery at <33 wks’ gestation (External validation)	0.80–0.93	90%
Encompass^TM^ [26,27,28]	≥35 yo, no high-risk conditions for preeclampsia, test diagnosis of placental-associated hypertensive disease ≥18 yo, no high-risk conditions for preeclampsia, test diagnosis of placental-associated hypertensive disease	18	All preeclampsia (Internal validation) Preeclampsia with delivery at ≤35 wks (Internal validation)	0.88 0.83	90 81%

## Data Availability

The data presented in this study are available on request from the corresponding author due to privacy restrictions.
